# The Prescriber’s Pharmacopia

**Published:** 1847-02

**Authors:** 


### ARTICLE XI.

*The Prescriber's Pharmacopia.* Published by S. S. & W. Wood, N. Y.

The above is the title of a small volume which has reached its third edition. It should be in the pocket of every young physician, containing, as it does, the most received prescriptions of the London and U. S. Pharmacopia's, arranged alphabetically under their appropriate heads. It will enable him promptly to select the one best adapted to any case in hand, without the necessity of a reference to an elaborate dispensatory. Moreover, time is often not afforded for such reference when the necessity for a prescription exists,—it is made from the physician's own memory of the effect of the substances entering into its composition, and he is liable to associate incongruous or incompatible articles. This unpretending little volume contains far more useful matter than many that are larger. Its assumed standard doses of many articles are much smaller than extended experience in their use warrants, when a decided effect is desired. G. N. F.

